# A Profound Membrane Reorganization Defines Susceptibility of *Plasmodium falciparum* Infected Red Blood Cells to Lysis by Granulysin and Perforin

**DOI:** 10.3389/fimmu.2021.643746

**Published:** 2021-05-19

**Authors:** Maria Andrea Hernández-Castañeda, Marilyne Lavergne, Pierina Casanova, Bryan Nydegger, Carla Merten, Bibin Yesodha Subramanian, Patricia Matthey, Nils Lannes, Pierre-Yves Mantel, Michael Walch

**Affiliations:** Department of Oncology, Microbiology and Immunology, Faculty of Science and Medicine, University of Fribourg, Fribourg, Switzerland

**Keywords:** blood-stage malaria, pore forming proteins (PFPs), perforin, granulysin, plasma membrane, cholesterol, phosphatidylserine (PS)

## Abstract

Malaria remains one of the most serious health problems in developing countries. The causative agent of malaria, *Plasmodium* spp., have a complex life cycle involving multiple developmental stages as well as different morphological, biochemical and metabolic requirements. We recently found that γδ T cells control parasite growth using pore-forming proteins to deliver their cytotoxic proteases, the granzymes, into blood residing parasites. Here, we follow up on the molecular mechanisms of parasite growth inhibition by human pore-forming proteins. We confirm that *Plasmodium falciparum* infection efficiently depletes the red blood cells of cholesterol, which renders the parasite surrounding membranes susceptible to lysis by prokaryotic membrane disrupting proteins, such as lymphocytic granulysin or the human cathelicidin LL-37. Interestingly, not the cholesterol depletion but rather the simultaneous exposure of phosphatidylserine, a negatively charged phospholipid, triggers resistance of late stage parasitized red blood cells towards the eukaryotic pore forming protein perforin. Overall, by revealing the molecular events we establish here a pathogen-host interaction that involves host cell membrane remodeling that defines the susceptibility towards cytolytic molecules.

## Introduction

Malaria is a life-threating disease that claims the lives of more than 400 000 people annually (1). *Plasmodium* spp., the infectious causative agent of malaria, have a complex life cycle, including a liver and a blood stage in the human host. It is widely accepted that the asexual intraerythrocytic replication of the parasites during the blood stage causes clinical malaria with all the associated morbidity and mortality. The replication cycle starts with the release of merozoites into the blood stream. The merozoites quickly invade uninfected red blood cells (RBCs) where they differentiate into ring forms that further grow into trophozoites. In the subsequent schizont stage, the parasites nucleus divides several times giving rise to daughter merozoites that egress the infected RBC to enter a new replication cycle (2).

During the development of the asexual parasite, the volume of the RBC increases slightly (by ~17%) while the parasite develops into the trophozoite stage (3). However, at the same time the parasite occupation within the RBC volume changes from initially 4 to 80% (4). There is an enormous demand for lipids due to parasite growth inside the host cell and subsequent replication (5, 6). In addition, if cholesterol, the only component known to be essential for microdomains/detergent-resistant membranes formation (7), is depleted from trophozoite-infected red cells, the parasite is expelled from the vacuole, suggesting that in the course of intracellular growth, cholesterol is essential to maintain infection (8). Despite the high lipid demands, the parasites are not capable to synthesize fatty acids or cholesterol *de novo* (9, 10). Therefore, the parasites extract these lipids directly from the serum or from the membranes of the host (11), leading to an inwards flow of lipids, including cholesterol (12).

In uninfected RBC, phosphatidylcholine and sphingomyelin are mainly located in the outer leaflet, while phosphatidylethanolamine, phosphatidylserine (PS) and phosphatidylinositol are mostly found in the inner leaflet of plasma membranes. Cholesterol is equally distributed in both membrane layers (13). PS plays a role in the cytoskeleton reorganization during the invasion of the parasite (14). Importantly, exposure of PS to the outer membrane leaflet was demonstrated in *Plasmodium-*infected RBCs (15, 16), with a potential role in cytoadherence (17). Marked alterations in the phospholipids content and composition of the plasma membranes of the late stage infected RBC (iRBC) compared to uninfected RBC were previously reported. Several groups notably observed an increased proportion of phosphatidylcholine and phosphatidylinositol in the plasma membrane of iRBC whereas the proportion of sphingomyelin and, in particular, cholesterol was decreased (5, 11, 12).

Cytotoxic lymphocytes (CTL) kill pathogen-infected cells and the intracellular pathogens by the release of their cytotoxic granule content. CTL can also limit the infectious spread of extracellular bacteria *via* the attenuation of their virulence (18). Target cell death and pathogen elimination is mediated by cytotoxic serine proteases, the granzymes (Gzms), that are delivered into the target cell by the pore forming protein perforin (PFN) (19, 20). Cytotoxic granules of some mammals contain another cytolytic protein, granulysin (GNLY) that preferentially targets prokaryotic cholesterol-poor membranes, such as of bacteria (21, 22). GNLY was shown to cooperate with PFN to deliver the Gzms into certain intracellular pathogens, such as *Listeria monocytogenes* (23) or *Trypanosoma cruzi* (24). We have recently discovered that the particular subset of cytotoxic lymphocytes, bearing the γδ T cell receptor, specifically recognizes, binds and reduces *P. falciparum* viability in a granzyme (Gzm)-dependent manner when delivered into the infected RBCs (iRBCs) by PFN or GNLY in a stage specific manner (25). PFN promoted transfer of granzyme B (GzmB) predominantly in early stages of *P. falciparum*- iRBCs and uninfected RBCs, while GNLY exclusively shuttled GzmB into late stage iRBCs and merozoites, independently of PFN. Here, we aimed to decipher the molecular determinants in the parasite surrounding membranes that define the susceptibility towards immune effector molecules.

## Materials and Methods

### GNLY, GzmB and PFN Purification

Cytotoxic granules were purified from the human NK cell line YT-Indy as described (26, 27). In brief, YT Indy cells were grown in RPMI + 10% FBS (Gibco) + antibiotics to about 3x10^9^ cells and harvested by centrifugation. The cellular pellet was resuspended in ice-cold relaxation buffer (10 μM PIPES, 0.1 M KCl, 3.5 μM MgCl_2_, 1 mM ATP, 1.25 μM EGTA, 0.05% BSA, pH 6.8) before disruption in a nitrogen cavitation bomb at 35 bar at 4°C for 15 minutes. Unbroken cells and nuclei were removed by centrifugation (400 × *g* for 7 minutes at 4°C) and the post-nuclear supernatant was spun (15000 × *g* for 15 minutes at 4°C) to obtain the granule pellet. The granule proteins were extracted by repeated freeze/thaw in extraction buffer (1 M NaCl in 20 μM Na-acetate, pH 4.5 containing 2 μM EGTA; 1 μl per 3x10^9^ cells) and the resulting lysate was centrifuged (15000 × *g* for 10 minutes at 4°C) and filtered (0.45-µm syringe filter, Millipore).

For GzmB and GNLY purification, the granule extract was diluted in S column buffer (50 µM bisTris, 150 μM NaCl, pH 7.4, filtered), load on a S column (HiTrap SP HP; GE) and eluted using a linear NaCl gradient (to 1M). GzmB activity was determined colormetrically using N-Ac-IEPD-pNA (Sigma) in a microplate reader (Synergy H1, Biotek). GNLY was identified by SDS-PAGE analysis, concentrated and the activity was assessed by standard CFU assays (28). GzmB was partly labelled using Alexa Fluor™ 488 Microscale Protein Labeling Kit (Invitrogen) as done recently (18).

For PFN purification, the granule extract was buffer exchanged to PFN-IMAC buffer (1 M NaCl, 20 μM HEPES, 10% betaine, pH 7.4, filtered) using an Econo-Pac 10 DG column (BioRad), then load on a cobalt-IMAC column (HiTrap IMAC HP 5 μl; GE) and eluted using a linear imidazole gradient (to 500 μM). The hemolytic activity of PFN was determined by hemolysis assays (see below). One hemolytic unit is defined as the amount of PFN to yield 50% lysis in this assay.

### Parasites

Cultures of the 3D7 strain *P. falciparum* were used in the experiments. Parasites were cultured in human A^+^ red blood cells (obtained from healthy volunteers), in malaria culture medium (MCM) composed of RPMI-1640 (25 μM HEPES, low bicarbonate, no glutamine, Sigma) supplemented with 1% human serum, Albumax II (Gibco), gentamicin (Sigma), 20% glucose and hypoxanthine as previously described (29, 30). The parasites were maintained at 37°C in 5% C0_2,_ 5% 0_2,_ 90% N_2_ and hematocrit adjusted to 4%.

### Stage Specific Parasite Enrichments

To identify altered conditions during maturation of parasites in RBCs, ring- and late stage iRBCs were enriched using magnetic separation techniques (31). After rinsing column (MultiMACS LS) with warmed 37°C malaria culture medium, parasite cultures were loaded on column and flow-through was collected simultaneously. The column was rinsed twice with 5mL MCM after the isolation was carried out. To achieve the same cell amount, the resultant flow-through was diluted 1:5000. To elute the column-bound late stage parasites, the column was removed from the magnetic support and rinsed with further 5 mL MCM. This eluent and the resultant flow through were then centrifuged (500 x *g*, 3 minutes). The supernatant was discarded, and pellets resuspended in 1 mL cell buffer and centrifuged again. The purified and flow-through cells were assessed by confocal microscopy; blood smears were prepared and stained with Hoechst.

Alternatively, percoll gradient purification was used to enrich for trophozoites and schizonts according to (32).

### Calimycin and Methyl-β-Cyclodextrin Treatment for Phosphatidylserine Exposure and Cholesterol Depletion, Respectively

RBC were washed once with PBS and then resuspended at 0.5 HCT in 40 μM Hepes (pH=7.4), 100 μM NaCl, 5 µM glucose +/- 2 μM CaCl_2_ before treatment with 1 µM calimycin (A 23187; Sigma) for 3 hours at 37°C. After the treatment, RBCs were subjected to Annexin V staining or hemolysis assays (see below).

RBCs were treated with indicated concentrations of methyl-β-cyclodextrin (Sigma) in RPMI for 2 hours at 37°C, then washed with PBS and subjected to filipin staining or haemoglobin release assays (see below).

### Annexin V/Propidium Iodide Stainings

*P. falciparum*-iRBC from unsynchronized cultures were subjected to either 65% or 75% Percoll for the enrichment of RBC that primarily contained schizonts or trophozoites, respectively. Mixed stages or purified iRBC were washed with PBS and the pellet was suspended in 100 µl Annexin V binding buffer solution (1x10^6^ cells/mL) containing FITC-annexin V (Miltenyi Biotech GmbH, Bergisch-Gladbach, Germany, 1:20) and Draq5 (BD, Bioscience, 1:500). Draq5 is a far-red DNA stain for live cells and therefore an ideal counterstain for the green FITC-annexin V for cytometry settings without a UV laser. The mixture was incubated for 15 minutes at RT in the dark. 400 µL of annexin V binding buffer was added to each tube and samples were analysed by flow cytometry on an Accuri C6 cytometer (BD Biosciences, San José, CA) using the FlowJo software (Data Analysis Software, Ashland, OR). For some experiments, annexin V stained parasites were counterstained with propidium iodide and Hoechst (both Sigma, 1μg/µl) and analyzed by confocal microscopy.

Alternatively, mixed stage cultures, calimycin treated RBCs or MACS purified late stages were stained with FITC labelled annexin V in 40 μM Hepes (pH=7.4), 100 μM NaCl, 5 μM glucose and 2 μM CaCl_2_ for 60 minutes on ice before fixation, nuclear staining with Hoechst and analyzed by confocal microscopy (Leica SP5). Fluorescence of calimycin treated cells was also assessed by plate reader (488/535 nm, Synergy H1, Biotek).

### Hemoglobin or BCECF Release Assays

Serial dilutions of GNLY, GzmB and PFN as indicated in assay buffer (40 μM Hepes, pH 7.4, 50 μM NaCl, 5 μM glucose) were performed in 96-well plate (round bottom) before adding an equal volume of RBCs washed and diluted to HCT 0.4% in cell buffer (assay buffer + 4 μM CaCl_2_). Samples were incubated for 15 minutes at 37°C and centrifuged for 4 minutes at 500 x *g*.

To measure hemoglobin release, 80 µL of supernatant was transferred to a 96-well plate (flat bottom) and the hemolytic activity was measured in a plate reader at 405nm (Synergy H1, Biotek). The specific lysis was calculated as the percentage of triton lysis minus the spontaneous release. 20 µl aliquots of the supernatants were additionally subjected to western blot analysis (see below).

To monitor plasma membrane integrity according to (33), MACS purified or flow-through iRBCs were labelled with 2’,7’-Bis-(2-Carboxyethyl)-5-(and-6)-Carboxyfluorescein, Acetoxymethyl Ester (BCECF-AM, Sigma, 1 µg/μl) in MCM for 30 minutes, then washed twice and resuspended in cell buffer. After indicated times, the samples were spun (450 x g, 3 minutes) and the supernatants were subjected to plate reader readings (FITC settings, Synergy H1), normalized to triton lysis as above.

### Red Blood Cell Fractionation – Western Blots

Western blot analysis was used to measure the release of RBC and plasmodial proteins from treated cells. 20 µL of residual supernatant from the hemolysis assays were transferred to microcentrifuge tubes and 5 µL Laemmli-buffer (5x) were added. The samples were then denatured at 95°C for 5 minutes. Substrates were separated by SDS-PAGE and then transferred to PVDF membrane by semi-dry WB. The membrane was washed with TBS-Tween-0.05% (TBS-T) solution and then blocked for 30 minutes at RT in blocking buffer (TBS-T + 1% BSA; Sigma). The membranes were incubated with primary rabbit anti-*Plasmodium falciparum* LDH antibodies (Sino Biological) over night at 4°C.

The membranes were washed with TBS-T at RT and then probed with secondary anti rabbit HRP antibodies (R&D Systems) for 45 minutes at RT. After TBS-T washes, chemiluminescence solution (ECL) was added to the membrane and was then exposed to gel imaging system (Syngene, G:BOX Chemi XRQ).

After the imaging, the membranes were washed in TBS-T and then subjected to the Abcam mild stripping protocol (https://www.abcam.com/ps/pdf/protocols/stripping%20for%20reprobing.pdf). Stripped membranes reprobed with rat anti-human GAPDH (Invitrogen) antibodies overnight at 4°C, then subjected to anti-rat HRP (R&D Systems) and imaged again.

### Assessment of Cholesterol Content in RBC Membranes

#### SLO-AF488 Staining

Streptolysin O (SLO) binding to membrane cholesterol was assessed according to (34). SLO was conjugated to AF488 using Alexa Fluor™ 488 Microscale Protein Labeling Kit (Invitrogen) following manufacturer`s recommendations. Mixed iRBCs were incubated with 2 U/mL SLO-AF488 in 40 μM Hepes (pH 7.4), 100 μM Nacl, 5 μM glucose for 60 minutes on ice (to minimize cell lysis) in the dark. Cells were washed twice with PBS, labelled with Hoechst during PFA fixation and then analyzed by confocal microscopy (Leica SP5).

#### Filipin Staining

****MACS purified or cholesterol depleted and fixed RBCs were resuspended in 500µl of PBS. 12.5µl of Filipin (Sigma) was added to the samples and incubated for 1h at RT in the dark. Subsequently the cells were washed twice with PBS and placed in a flat-bottom 96 well plate. The filipin intensity was measured in a plate reader at 350/480 nm and normalized to RBC density at 650 nm (Synergy H1, Biotek).

### Statistics

Significant differences were determined alternatively by Microsoft Excel (Microsoft Corporation, Redmond, WA) and GraphPad Prism (GraphPad Software, San Diego, CA). All experiments were performed in triplicates and were at least twice independently repeated. Data are presented as means ± SEM. Comparisons between the different groups were performed with two-tailed unpaired Student`s t tests (using Microsoft Excel). P values are indicated in the Figures. *P* values of less than 0.05 were considered significant.

## Results

### An On-Going *Plasmodium falciparum* Infection Renders Parasitized RBCs Susceptible to GNLY While They Become Simultaneously Resistant to PFN Lysis

**Figure 1:**
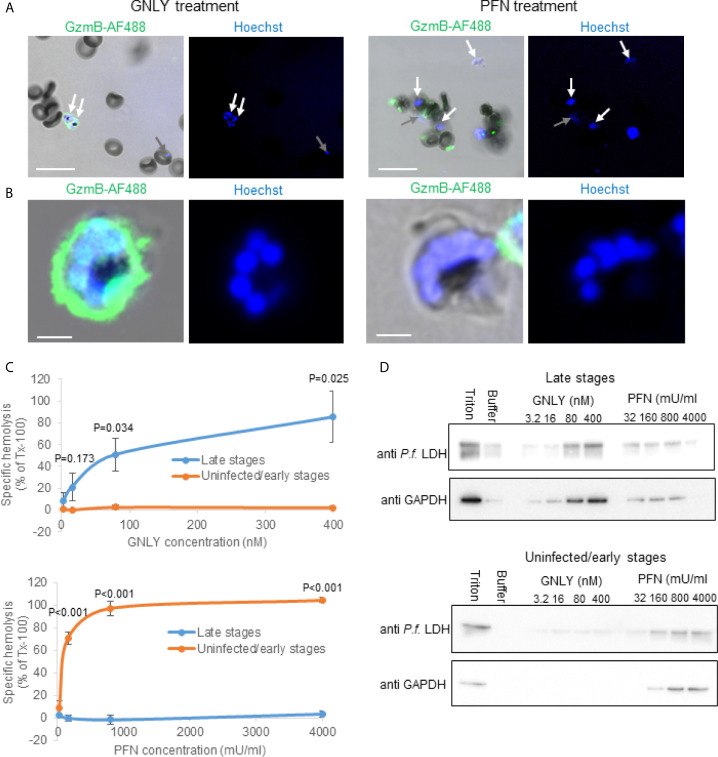
*Plasmodium falciparum* infections render parasitized RBCs susceptible to GNLY while they become simultaneously resistant to PFN lysis. **(A, B)**, non-synchronized iRBCs cultures were treated with Alexa Fluor 488-labeled GzmB (0.4 μM) in combination with GNLY (0.4 μM) or with PFN (500 mU/μl) for 20 minutes before fixation and assessment by confocal microscopy. Nuclei were stained with Hoechst and are indicated in blue. Late stage parasites are indicated with white arrows and early stage parasites with gray arrows. Scale bars are 10μm in **(A)** and 2μm in **(B)** MACS-purified (late stages) and flow-through cells (uninfected and early stages) were treated with indicated concentrations of GNLY and PFN for 15 minutes at 37°C before centrifugation to separate the cellular pellet from the supernatant. The supernatants were subjected to optical densities measurement at a wavelength of 405 nm to assess hemoglobin release **(C)** and to immunoblot analysis to monitor release of plasmodial LDH and RBC GAPDH **(D)**.

One of the starting points into this study was the observation that the prokaryotic membrane disrupting protein, GNLY, was capable to deliver labelled GzmB exclusively into late stage iRBCs to inhibit their growth (25). On the other hand, PFN, that is structurally related to cholesterol-dependent cytolysins (35), delivered GzmB in uninfected RBCs and all stages of iRBCs with an assumed exception of very late stages (i.e. schizonts) (**Figures 1A, B**). A thorough quantification strongly suggested a stage-dependent inverse activity of these cytolysins (25), however, it was impossible to draw a firm conclusion based on microscopical assessments alone.

To assess stage-dependent PFN and GNLY activity on *Plasmodium*-infected RBCs more quantitatively, MACS-purified (late stages) and flow-through (uninfected and early stages) were treated with different concentrations of these effector molecules for hemoglobin release (**Figure 1C**) and fractionation assays (**Figure 1D**). As there was some concern about the structural integrity of the MACS purified late stage iRBCs in the hypotonic buffer condition of these experiments, we analyzed the purified cells by live cell high-resolution confocal microscopy (**Figure S1**). The MACS purified cells did not reveal obvious signs of structural disintegration after up to 60 minutes of scanning in hypotonic buffer conditions. In the fractionation assays, the iRBC supernatant after the treatment was screened for plasmodial lactate dehydrogenase (LDH) as well as RBC GAPDH by western blot to assess protein leakage from different intracellular compartments. While the detection of plasmodial LDH indicates rupture of all membranes surrounding the parasite (the parasite plasma membrane, the parasitophorous vacuole membrane and the RBC membrane), GAPDH only indicates the rupture of the RBCs. Although there was some spontaneous protein release from the more fragile iRBCs (in particular visible in the LDH blots), both hemoglobin release and fractionation assay clearly demonstrated that GNLY almost exclusively lysed MACS purified late stages confirming the imaging results. PFN demonstrated inverse behavior, causing lysis in the MACS flow-through cells (uninfected and early stages) while late stage iRBCs were remarkably resistant to PFN lysis.

### Cholesterol Is Depleted in Late-Stage iRBCs

**Figure 2:**
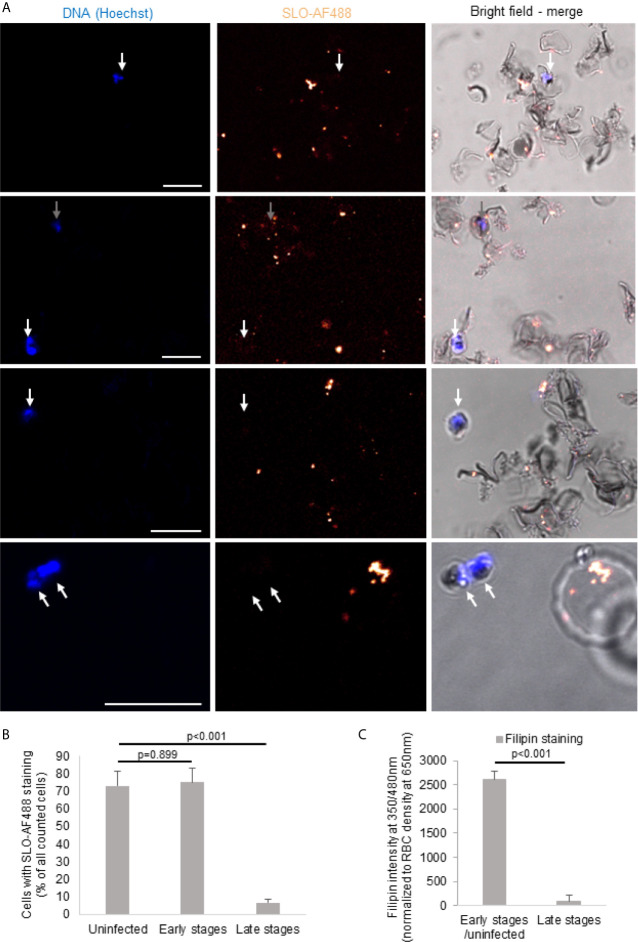
Cholesterol is depleted of late-stage iRBCs. Non-synchronized iRBCs were treated with Alexa Fluor 488-labeled streptolysin O (SLO-AF488; 2 U/μl) for 60 minutes on ice before fixation and assessment by confocal microscopy. Nuclei were stained with Hoechst. Scale bars are 10μm. Four representative images from three independent experiments are shown in **(A)**. The white arrows indicate the late stage iRBC (not stained with the SLO-AF488) and the gray arrows show the early stage iRBC (stained with the SLO-AF488). The quantification (n>40 for each stage form in every independent experiment) of the three independent experiments is presented in **(B)**. **(C)** iRBCs were MACS purified to enrich for late stages before filipin staining and fluorescence measurement by plate reader and compared to MACS flow-through cells (early stages and uninfected). Averages +/- SEM of three independent experiments are shown. P values of differences, calculated by Student`s t test, are indicated.

During maturation of the parasite, the RBC membrane lipid composition is modified (11). However, there is conflicting evidence in the literature to what extent this modifications also lead to the depletion of cholesterol in the parasite surrounding membranes, including the RBC plasma membrane (12, 36–38). Therefore, we set out to indirectly measure the cholesterol content in the plasma membrane of iRBCs of different stages by the binding capacity of the cholesterol-binding cytolysin streptolysin O (SLO) using an adapted protocol from (34). As observed in representative images (**Figure 2A**) and confirmed by quantification (**Figure 2B**), bound SLO was found on almost all uninfected RBC and early stage iRBCs (gray arrows) (**Figures 2A, B**). However, late stage iRBCs were virtually excluded from SLO binding suggesting a complete depletion of SLO (white arrows).

These results of SLO binding were confirmed by filipin staining of MACS-purified late stage iRBCs as compared to the flow-through cells (**Figure 2C**). Filipin, a naturally fluorescent antibiotic with high binding affinity to cholesterol (39), was significantly decreased on late stage iRBCs as assessed in fluorescence readings. Combined, these data indicate an efficient cholesterol depletion of the iRBC plasma membrane in the course of the infectious cycle.

### Cholesterol Depletion Increases Susceptibility of RBC Membranes to GNLY and, Surprisingly, to PFN Lysis

We have previously shown that GNLY predominantly disrupts cholesterol poor membranes, such as of prokaryotes (40). In order to demonstrate the effect of cholesterol depletion in the setting of RBCs, we mimicked late stage parasites by depleting cholesterol of uninfected RBCs using methyl-β-cyclodextrin followed by treatment with GNLY and PFN. Methyl-β-cyclodextrin dose-dependently depleted cholesterol from RBC membranes as indicated by filipin staining (**Figure S2A**). The depletion was almost complete at the concentration of 7.5 μM, indicated in low filipin fluorescence intensity, very similar to that in MACS-purified late stage iRBCs (**Figure 2C**). The efficiency of the lysis was assessed by hemoglobin release assays. As controls targeting cholesterol-poor and rich RBC membranes, we used respectively the human antimicrobial peptide, LL-37 (41), and the cholesterol dependent cytolysin SLO (42).

**Figure 3:**
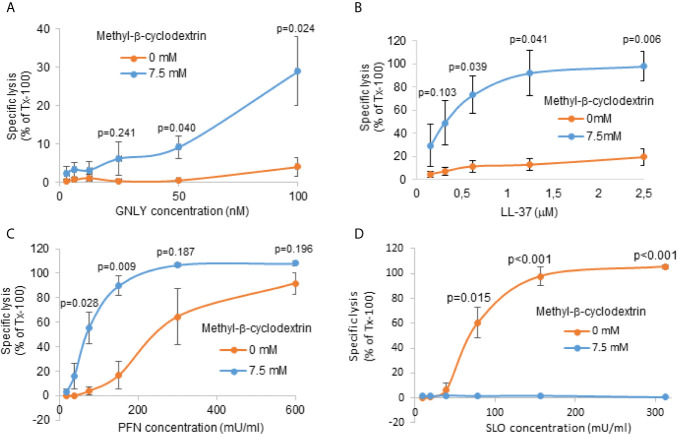
Cholesterol depletion increases susceptibility of RBC membranes to GNLY and to PFN lysis. RBCs were pre-treated with 7.5 μM methyl-β-cyclodextrin for 2 hours at 37°C to deplete cholesterol. Depleted cells were then treated with indicated concentrations of GNLY **(A)**, LL-37 **(B)**, PFN **(C)** or SLO **(D)** for 15 minutes at 37°C before hemoglobin release was assessed by plate reader at 405 nm. Averages +/- SEM of specific lysis in three independent experiments are shown. P values of differences between treatment conditions, calculated by Student`s t test, are indicated.

Treatments with both GNLY and LL-37 led to significantly more lysis in cholesterol depleted RBC membranes (**Figures 3A, B**), supporting the hypothesis that the cholesterol content in the iRBC membrane is crucial for the interaction and activity of antimicrobial proteins. Though also highly positively charged, GzmB alone did not affect cholesterol-depleted RBCs (**Figure S2B**), consistent with our previous results that for GzmB uptake into late stage parasites GNLY is necessary (25). PFN promotes transfer of GzmB in cholesterol-rich mammalian cells and is structurally related to the bacterial cholesterol-dependent cytolysins (43); however, early studies on PFN revealed indeed pore formation in absence of cholesterol (44, 45). Strikingly, treatment with PFN led to significantly more lysis in cholesterol-depleted membranes (**Figure 3C**) compared to non-treated RBCs. On the contrary, RBCs treated with SLO did not show any lysis in the cholesterol-depleted membranes (**Figure 3D**), but displayed efficient lysis in the control samples, consistent with the strict cholesterol-dependency of this pore-forming cytolysin. These results demonstrate that, although PFN promotes transfer of GzmB in cholesterol-rich mammalian cells, in this experimental setting, it acted independently of cholesterol, therefore, raising the question of what, if not cholesterol-depletion, rendered late stage iRBCs resistant to perforin lysis.

### *Plasmodium falciparum* Infection Triggers Exposure of Phosphatidylserine in Late Stage iRBCs

**Figure 4:**
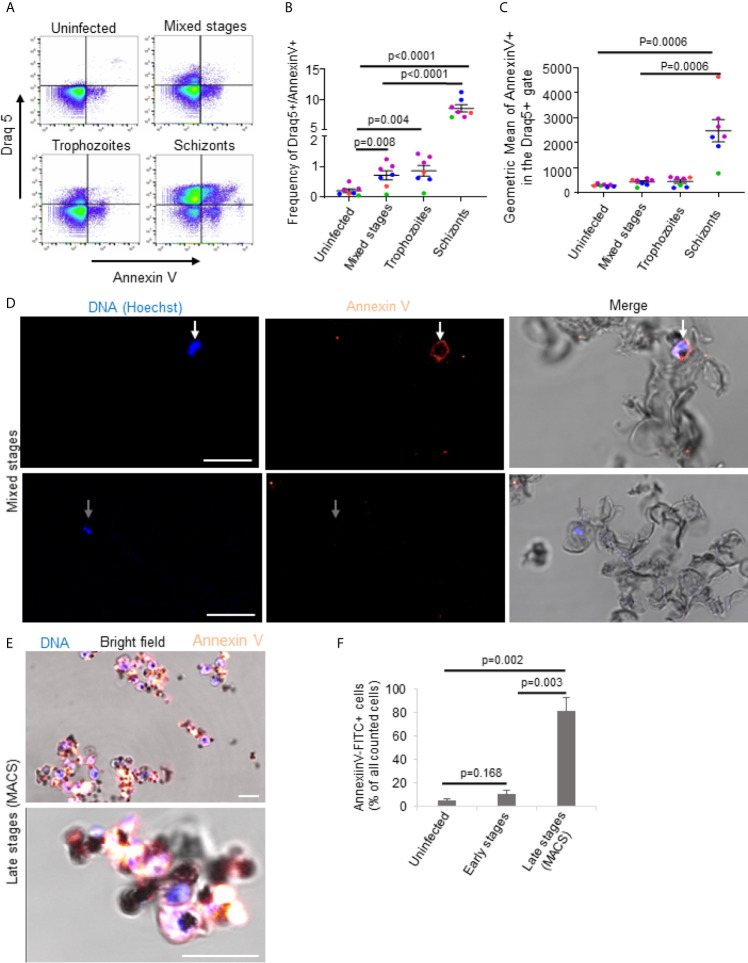
*Plasmodium falciparum* infection triggers exposure of phosphatidylserine in late stage iRBCs. Uninfected erythrocytes, mixed stages, 75% Percoll enriched trophozoites and 65% Percoll enriched schizonts were incubated with annexin V-FITC and Draq5 for 15min at RT and subjected to flow cytometry analysis. A representative FACS dot plot of annexin V versus Draq5 staining is shown in **(A)**. Averages and values of single experiments represented by dots in different colors indicating frequencies of Draq5+/Annexin V+ are depicted in **(B)** and indicating geometric mean of annexin V in Draq5 + are presented in **(C)**. Mixed **(D)** or MACS-purified late stage iRBCs **(E)** were stained with Annexin V-FITC for 60 minutes on ice before fixation and analysis by confocal microscopy. Nuclei were stained with Hoechst. Representative images of three independent experiments are shown in **(D, E)**. In **(D)**, scale bars are 10μm. The quantification of the microscopy results and presentation as averages +/- SEM is shown in **(F)**. P values of differences between stages were calculated with the unpaired t-test **(B, F)** or Mann-Whitney test **(C)**. Independent experiments are represented in different colors and replicates by symbol in **(B, C)**.

Given the cholesterol-independent activity of PFN, the question remained, what, if not cholesterol depletion, rendered late stage iRBCs resistant towards lysis by PFN. A recent study revealed that cytotoxic T cells protect themselves from PFN autolysis in the immunological synapse by the exposure of phosphatidylserine (PS) (46). PS is a charged membrane phospholipid, which in intact cells is exclusively localized in the inner leaflet of the lipid layer. However, once cells undergo apoptosis or oxidative stress, PS molecules are exposed on the external surface of the cells (47). PS exposure was also found in higher frequencies in late stages iRBCs (48). To evaluate the differences in PS exposure in uninfected and infected RBCs, annexin V binding assays were performed. Annexin V is known to bind specifically to the polar head group of PS in the presence of calcium (32, 49). By using flow cytometry, we confirmed that annexin V-binding to RBCs correlated with developmental stage of the parasite (**Figures 4A–C**). While there was only a slight increase of annexin V staining frequency and intensity in mixed stage and pure trophozoites cultures, purified schizont cultures demonstrated a highly significant enhanced PS exposure. These cytometry results were corroborated by confocal microscopy of mixed stage cultures (**Figure 4D**) and, particularly, of MACS purified late stage cultures, in which almost all the cells displayed a bright annexin V staining (**Figures 4E, F**). To exclude that annexin V could bind ruptured membranes in late stage parasites, we performed annexin V stainings in combination with propidium iodide (PI), known to be excluded from intact cells (**Figure S3**). Though the cells efficiently excluded PI early in the experiments, there was a certain tendency for PI positive cells of low intensity after 60 minutes (**Figure S3A**). However, the PI staining intensity was clearly increased in saponin treated cells (**Figure S3B**). In addition, we labelled MACS purified late stages and flow through cells with the fluorescent dye BCECF-AM, known to be trapped in living cells with intact membranes (28). Indeed, we did not detect significant differences in the release of fluorescence from late versus early stages in the course of the experiment (**Figure S3C**). These results clearly indicate elevated exposure of PS in the outer surface of the late stage iRBC, which may be the cause for the increased resistance to PFN lysis.

### Exposure of Phosphatidylserine in the Outer RBCs Plasma Membrane Leaflet Mediates Resistance to PFN Lysis

**Figure 5:**
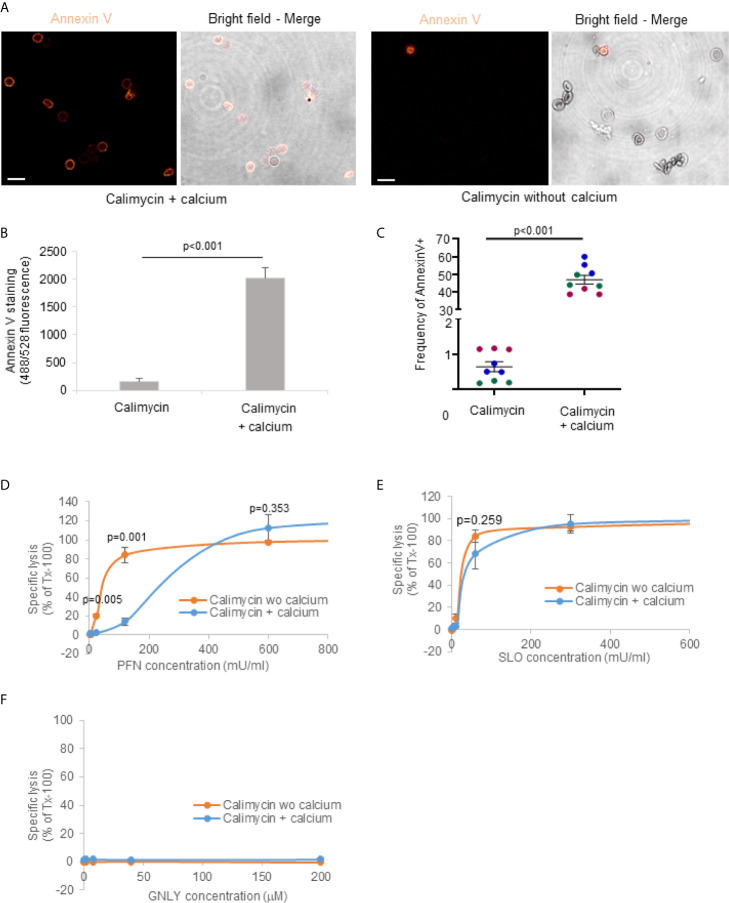
Exposure of phosphatidylserine in the outer RBCs plasma membrane leaflet mediates resistance to PFN lysis. RBCs were pre-treated with 1 μM calimycin +/- 2 μM calcium for 3 hours at 37°C, then washed with PBS and stained with Annexin V-FITC for 1 hour on ice for confocal microscopy **(A)** or treated with indicated concentrations of PFN **(D)**, SLO **(E)** or GNLY **(F)** for 15 minutes at 37°C before hemoglobin release assay. Averages +/- SEM of the fluorescence of annexin V stained cells assessed by plate reader is demonstrated in **(B)**. Averages and values of single experiments represented by dots in different colors indicating the frequency of annexin positive cells assessed by flow cytometry are shown in **(C)**. In **(A)**, scale bars are 10μm. P values of significant differences were calculated with unpaired t-test. wo, without.

In order to demonstrate a direct link between PS externalization and increased resistance to PFN lysis, we treated RBCs with calimycin, an ionophore highly selective for Ca^2+^ (50). Exposure of RBCs to 1 µM calimycin, in presence of Ca^2+^, significantly increased annexin V binding to RBCs as demonstrated by confocal microscopy (**Figure 5A**), fluorescence readings by plate reader (**Figure 5B**) and flow cytometry (**Figure 5C**). In contrast, cells treated in absence of Ca^2+^ did not show increased fluorescence signal and lower frequency of annexin V. These results indicated that calimycin treatment in presence of Ca^2+^ efficiently triggers the translocation of PS in the outer leaflet of RBCs. Importantly, pre-treatment of RBCs with calimycin and Ca^2+^ significantly decreased the susceptibility to PFN lysis (**Figure 5D**). The activities of SLO or GNLY were not altered under this condition (**Figures 5E, F**). These results indicated a PFN inhibitory effect of PS exposure on RBC membranes.

## Discussion

All symptoms of clinical malaria and in particular its most severe complication, cerebral malaria, are caused by the exponential growth of the parasite during the blood stage, subsequent cytoadherence and sequestration of iRBCs at critical sites in the microvasculature as well as overreactivity of the pro-inflammatory immune response (51–53). Therefore, to prevent malaria-associated morbidity and mortality tight control of parasitemia is essential. We previously demonstrated, and it was very recently independently confirmed (54), that the lymphocyte subset bearing the γδ T cell receptor contributes critically to the inhibition of parasite growth in the blood phase. The inhibition of late stage parasite depended on the expression of GNLY and GzmB but was independent of PFN. The molecular determinants of this differing susceptibility to lymphocytic cytotoxic effectors were undefined and was the very question of the present study.

The lifecycle of malaria parasite, in both the mammalian host and the mosquito vector, consists of multiple developmental stages (55). *P. falciparum* infection completely reorganizes the membrane compartment of the host RBC, including the modification of the RBC plasma membrane, parasitophorous vacuole membrane as well as additional membrane structure, such as a tubulovesicular network and the Maurer`s cleft (12). Though the parasite lacks the machinery to synthesize fatty acids and cholesterol, the lipid content of erythrocytes increases approximately six-fold during the latest stage of infection (56). As the development inside the RBC continues, the structural organization and stability of the lipid bilayer is altered by a constant inwards flow of various lipids, including cholesterol (12). However, it was still unclear how these changes in the RBC membrane composition impact on the susceptibility and sensitivity towards cytotoxic T cell attack. Because the parasite nourishes on cholesterol, we hypothesized that the decrease in cholesterol content in the outer membrane compartments renders late stage iRBCs more susceptible to GNLY (25, 40) and less susceptible to PFN. Indeed, cholesterol depletion from the RBC plasma membrane using methyl-β-cyclodextrin significantly increased the lytic activity of GNLY and LL-37 indicating that cholesterol is the defining factor for the susceptibility towards antimicrobial proteins. Furthermore, this mechanism explains the efficient killing of late stage parasites by GzmB and GNLY treatment. Despite PFN shares a common fold with cholesterol-dependent cytolysins (57, 58), in our experimental setting it acted in a cholesterol independent manner. There was more lysis in cholesterol-depleted RBCs. However, as absence of cholesterol increased lysis by PFN, cholesterol levels in the outer membranes can not explain that observed difference in PFN susceptibility of different iRBC stages. On the contrary, our results might suggest that PFN should more efficiently form pores in late stage iRBCs, because of the cholesterol depletion during the maturation of the parasite.

In addition, when we compared the susceptibility of purified late stages iRBCs towards GNLY and PFN as compared to uninfected and early stages iRBCs, we found that GNLY triggered release of hemoglobin and *Pf*LDH from late stage iRBCs, as did PFN from uninfected and early stage RBCs. The simultaneous release of a cytosolic parasite protein was surprising as it might indicate that these cytolysins were able to penetrate all the membrane systems surrounding the parasite, including the parasite plasma membrane. According to the inward lipid flow model (12), the cholesterol concentration is expected to increase in the inner membrane compartments, questioning a specific lytic effect of GNLY. It is also possible that lysis of the host cell membrane compromises the structural integrity of this obligatory intracellular pathogen. On the other hand, PFN might indeed sequentially penetrate all the membrane compartments to reach the early stage parasite.

In the erythrocyte, the choline-containing phospholipids, phosphatidylcholine and sphingomyelin, are present mainly in the outer monolayer, whereas the amino-phospholipids, phosphatidylethanolamine and phosphatidylserine (PS), are localized almost entirely in the inner monolayer. When the asymmetric distribution of phospholipids across the membrane is disrupted, as in disease or in aged red cells, the biophysical properties of the membrane itself, as well as the physiological attributes of the cell surface, are altered (59). PS is an important constituent of eukaryotic cellular membranes. On the plasma membrane of healthy cells, PS is found in the inner leaflet of the plasma membrane (60) and in endocytic membranes (61), but it is externalized in apoptotic cells. We hypothesized that PS exposure render late-stage parasites more resistant to PFN. CTLs are resistant to the action of their own cytotoxic molecules when delivered to the target cell. A proposed mechanism for this resistance is that exposed PS on CTLs sequesters and inactivates PFN (46). Indeed, increased PS exposure is found on late parasitized RBCs and forced PS externalization (by calimycin treatment) inhibited the lytic activity of PFN. In a broader view on our data, we conclusively demonstrated that PFN affects only early stage iRBCs. However, the overall importance of PFN for the inhibition of the dangerous exponential growth phase of *P. falciparum* in the blood is not clear yet as our (25) and other`s earlier findings (62) suggested that PFN is dispensable for cell-mediated *Plasmodium* elimination during the blood-phase.

Our experiments demonstrate a profound remodelling of the iRBC plasma membrane with an efficient depletion of cholesterol and a massive externalisation of PS in the course of the infection that defines the susceptibiltiy of GNLY and PFN lysis. These unique membrane features of late stage iRBCs might be useful in future studies to specifically target parasitized RBCs in the final hours before parasite egress and the continuation of the growth cycle. In his respect, it could be envisioned to treat blood stage malaria with antimicrobial peptide-like effector molecules, either in their free form or encapsulated in coated lipid nanocarriers for the specific delivery of the cargo into late stages iRBC. This technology is currently under investigation in our lab as well as in various related fields of medical research.

## Data Availability Statement

The raw data supporting the conclusions of this article will be made available by the authors, without undue reservation.

## Ethics Statement

We used for this study only human cells and blood products (serum) that we obtained from the Swiss Blood Bank in a completely anonymized fashion, therefore, this work was exempt from ethics approval. This exemption was confirmed on direct request by the cantonal ethics committee (request # CER-VD/Req-2018-00810) as Article 2al. 2c of the Human Research Act (HRA) states that the HRA does not apply to research performed on anonymized human material.

## Author Contributions

MW conceived the study. MW and P-YM conceptualized the study by providing the methodology and establishing the major assays. MH-C, ML, PC, BN, CM, PM, BS, NL, P-YM, and MW conducted the investigation, performed the experiments, and analyzed the data. MH-C, ML, and MW wrote and revised the manuscript. MW and P-YM supervised the study. All authors contributed to the article and approved the submitted version.

## Funding

This work was supported by the Swiss National Science Foundation (SNSF grant # 310030_169928 to MW), the Bangerter-Rhyner-Foundation (to MW and P-YM), and the Research Fund of the University of Fribourg (to MW).

## Conflict of Interest

The authors declare that the research was conducted in the absence of any commercial or financial relationships that could be construed as a potential conflict of interest.

## Abbreviations

BCECF-AM, 2’,7’-Bis-(2-carboxyethyl)-5-(and-6)-carboxyfluorescein, acetoxymethyl ester; CTL, Cytotoxic lymphocytes; DRAQ5, Deep red anthraquinone 5; FITC, Fluorescein isothiocyanate; GNLY, Granulysin; Gzm(s)/GzmB, Granzyme(s)/Granzyme B; (Pf)LDH, (*Plasmodium falciparum*) lactate dehydrogenase; LL-37, member of the cathelicidin family (peptide composed of 37 amino acids, including two leucines); MACS, Magnetic-activated cell sorting; PI, Propidium iodide; PFN, Perforin; PS, Phosphatidylserine; (i)RBC, (Infected) red blood cells; SLO, Streptolysin O.
