# Supplementary figures and images for: A Profound Membrane Reorganization Defines Susceptibility of Plasmodium falciparum Infected Red Blood Cells to Lysis by Granulysin and Perforin
Source: Front Immunol. 2021 May 19;12:643746. doi: 10.3389/fimmu.2021.643746 (PMC8170093; doi:10.3389/fimmu.2021.643746)

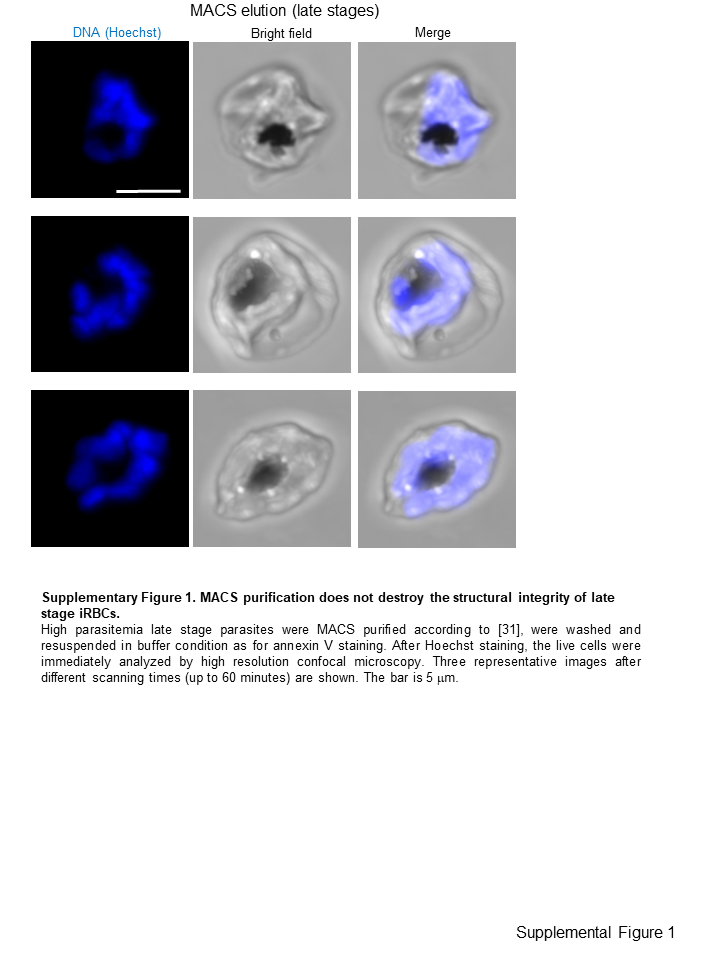

Supplement: Supplementary file 1 [file Image_1.tif]

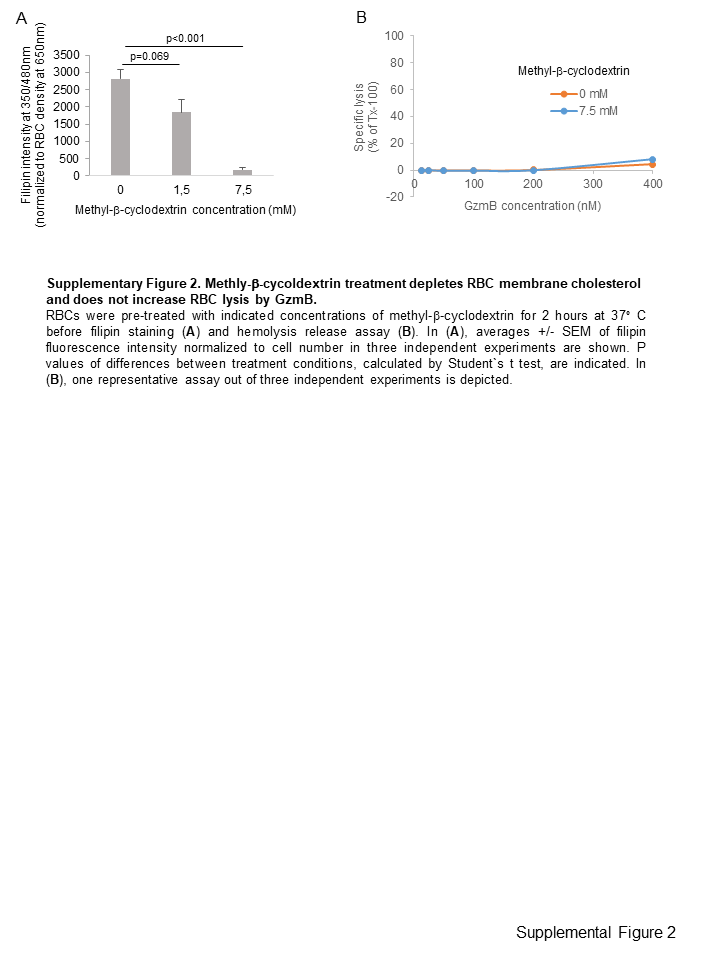

Supplement: Supplementary file 2 [file Image_2.tif]

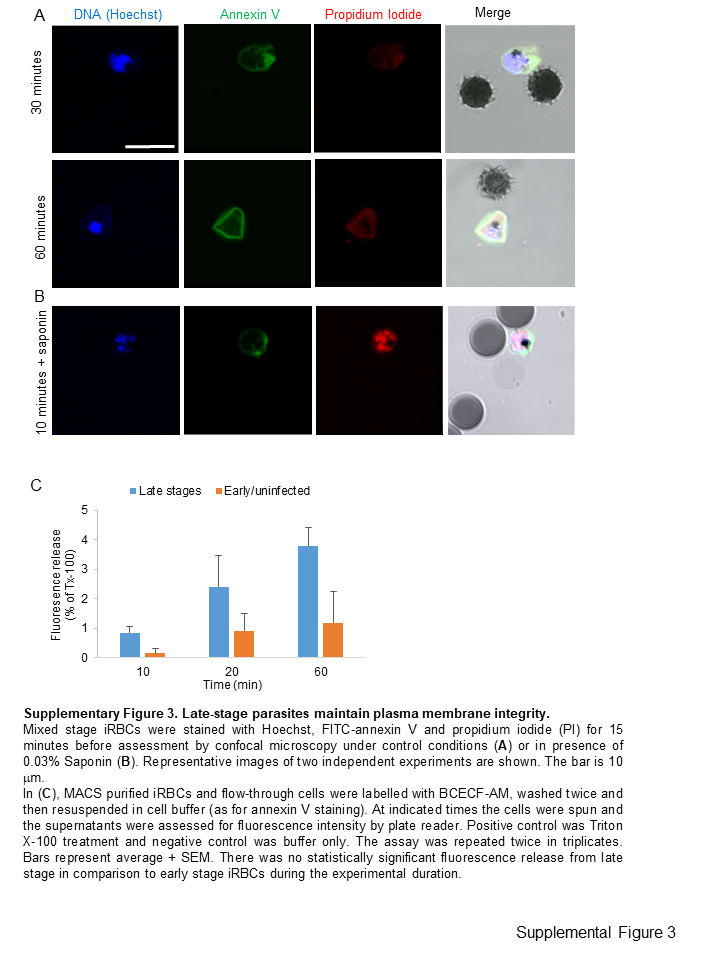

Supplement: Supplementary file 3 [file Image_3.tif]
